# Distribution Dynamics and Roles of Starch in Non-photosynthetic Vegetative Organs of *Santalum album* Linn., a Hemiparasitic Tree

**DOI:** 10.3389/fpls.2020.532537

**Published:** 2021-01-12

**Authors:** Xiu Ren Zhou, Ning Nan Zhang, Yi Min Zhao, Lei Dai, Da Ping Xu, Gui Fang Xu, Jing Tian

**Affiliations:** ^1^School of Life Science and Technology, Henan Institute of Science and Technology, Xinxiang, China; ^2^Research Institute of Tropical Forestry, Chinese Academy of Forestry, Guangzhou, China; ^3^Guangxi Botanical Garden of Medicinal Plants, Nanning, China

**Keywords:** stored starch, allocation dynamics, parasitic plants, Santalum album, soluble sugar, non-photosynthetic vegetative organs

## Abstract

Allocation dynamics of stored starch plays essential roles in the development and growth of trees. Previous studies focused on the dynamics and the characteristics of starch in autotrophic trees. However, although starch granules have been detected in the organs or tissues of some parasitic plants, studies on the allocation dynamics and roles of storage starch in them are limited. Therefore, we determined and estimated the allocation dynamics and roles of starch in *Santalum album* Linn., a hemiparasitic tree, using morphological and physiological methods. Our findings showed abundant starch in the stem and root of *S. album* at the early seedling stage. Although *S. album* seedlings attached to the host showed no significant changes in starch levels throughout the experiment, unattached and host-removed seedlings exhibited a gradual decrease in the starch content over time. When the starch content of unattached seedlings was less than 1%, they started to die. Starch accumulated to high levels in developing and active haustoria; however, starch levels were low in the inactive haustoria. The present study suggests that starch may provide energy to seedlings that have no host, allowing them to survive during the unattached phase, thus increasing their chance to attach to host roots by extending their survival duration. In addition, we speculate that storage starch is potentially involved in the development of haustoria and in the physiological processes of *S. album* related to the absorption and transportation of water and nutrients from its host.

## Introduction

Starch is the most critical long-term energy storage carbohydrate in plants. Under abiotic stress conditions, such as low/high temperature or drought, starch is degraded, generating osmotically active compounds and energy, which are used to ensure plant survival (Levitt, 1980; Guehl et al., 1993; Uemura and Steponkus, 2003; McDowell et al., 2008; Adams et al., 2009; Sala et al., 2010; Villar-Salvador et al., 2015). Deciduous trees use the starch stored in their stems and roots to promote sprouting and thickening of trunks and branches at the beginning of a growing season (Johansson, 1993; Bollmark et al., 1999; Kaelke and Dawson, 2005; Regier et al., 2010). In the Mediterranean, plants known as resprouters, which produce new shoots after a fire, contain much higher amounts of starch in their roots than seeders, which die after a fire and then re-establish from seeds (Bell et al., 1996; Bell and Ojeda, 1999; Verdaguer and Ojeda, 2002; Knox and Clarke, 2005). Perennial plants whose aerial parts are frequently destroyed or disturbed by biotic or abiotic stresses often store a greater starch content in their stems and roots than plants of the same species growing in an undisturbed habitat; the greater starch content can serve as an energy source for sprouting once the ability to assimilate carbon is lost (Hoffmann et al., 2004; Dietze et al., 2014). Storage starch plays a crucial role in ensuring the growth and development of plants in the absence of photosynthesis or under stress conditions (McDowell et al., 2008; Sala et al., 2010; Villar-Salvador et al., 2015).

To date, the role of starch allocation dynamics has mostly been investigated in autotrophic plants, such as poplar (Sauter, 1988; Johansson, 1993; Regier et al., 2010), oak (Barbaroux and Bréda, 2002), beech trees (Barbaroux and Bréda, 2002), and *Salix viminalis* (Bollmark et al., 1999). However, in parasitic plants, studies on the distribution dynamics and the function of storage starch are limited. Starch granules exist in the parenchyma cells of haustoria or tubercles of *Santalum album* (Zhang et al., 2012; Yang et al., 2014; Da Silva et al., 2016), *Cuscuta japonica* (Dawson et al., 1994; Lee, 2007), *Korthalsella* spp. (Fineran, 1996), *Orobanche* spp., *Phelipanche* spp., and *Striga gesnerioides* (Yoshida et al., 2016). Starch granules have also been detected in the tracheary elements of *Euphrasia cuneata*, *Lathraea clandestina*, and *Krameria grayi* (Fineran, 1985), as well as in flange-type cells in *Korthalsella* spp. (Fineran, 1996). In *C. japonica*, the abundance of starch granules in cortex cells increase upon the initiation of haustoria development (Lee, 2007). Starch stored in the tubercles of *Cistanche tubulosa*, *Orobanche cumana*, and *O. aegyptiaca* is assumed to support plant growth, flowering, and seed development (Joel and Losnergoshen, 1994; Chen et al., 2011). However, the distribution of starch in non-photosynthetic organs of parasitic plants and development-related changes in this distribution pattern remain unclear. In addition, the roles of starch in the growth of parasitic plants, and morphological and physiological evidence supporting these roles are largely unknown. Therefore, further research is needed to provide a comprehensive understanding of the allocation dynamics and the function of storage starch in parasitic plants.

*Santalum album* is the primary source of sandalwood, one of the most expensive woods because of its widespread application in the fragrance and incense industry. Previous studies indicate that starch granules are present in parenchyma cells located in different regions of the haustorium, for example, near the meristematic area and the collapsed tissue (Zhang et al., 2012; Yang et al., 2014; Da Silva et al., 2016). However, many aspects of the allocation dynamics and the roles of starch in stems, roots, and haustoria in *S. album* remain unclear. In this study, we focused on the qualitative visualization and the quantitative determination of starch allocation dynamics in non-photosynthetic organs (including haustoria) of *S. album* and on the potential roles of starch in this parasitic plant. The qualitative visualization of starch of stems, roots, and haustoria of *S. album* was analyzed by light microscopy and scanning electron microscopy. In addition, we quantified the amount of starch and soluble sugar in these organs and assessed the plant morphological characteristics, as well as sap flow, net photosynthesis, and transpiration rates, under different conditions.

## Materials and Methods

### Experimental Design and Treatments

This study was conducted at the field site of the Research Institute of Tropical Forestry (23°11′35.50″N, 113°22′45.60″E; 50 m a.s.l.), Chinese Academy of Forestry between January 2015 and March 2017. Field trials were laid out using a randomized complete block design with three blocks and three treatments. Each block was 54 m^2^ (18 m × 3 m) in size and was further divided into three plots of 18 m^2^ (6 m × 3 m). The three blocks were adjacent to each other, and the field was flat. No buildings or other plants disrupted the incoming sunlight in this field. Each plot contained 36 potted *S. album* seedlings, arranged at 1 m row-to-row spacing and 0.5 m plant-to-plant spacing (within each row). The potted seedlings were subjected to three treatments: US, where seedlings were unattached to hosts throughout the experiment; AS, where seedlings were attached to hosts throughout the study period; and RS, where seedlings were attached to hosts from February 2015 to November 2015, and the host was removed in early December 2015. These three treatments were randomly assigned to plots within a block such that each block consisted of one replicate of each treatment.

In early January 2015, the germination of *S. album* seeds was induced by gibberellic acid, and three germinated seeds were planted in each pot. After approximately 15 days, when all seedlings had emerged, only one seedling was retained in each pot. Then, 324 potted seedlings were randomly assigned to the three blocks in each plot. In early February 2015, two host (*Kuhnia rosmarnifolia*) seedlings were transplanted near each *S. album* seedling in the AS and RS treatment plots. In early December 2015, the hosts were removed from the RS plots.

### Planting Conditions

*Santalum album* seedlings were planted in pots (25 cm wide × 27 cm deep) filled with a mixture of sterilized pastoral soil and river sand (2:1, v/v). Each pot was fed weekly with 50 ml of all-nutrient solution, which was formulated according to Barrett and Fox (1997). The seedlings were watered to prevent drought stress. Weeds were removed from each pot to prevent their parasitization by *S. album*.

### Measurement of Sap Flow, Net Photosynthesis, and Transpiration Rates

In each plot, the sap flow, the net photosynthesis, and the transpiration rates of three randomly selected *S. album* seedlings were determined at 3-month intervals during a 2-year period (March 2015 through March 2017). Sampling was conducted in either sunny or slightly cloud weather. The sap flow rate was measured using a sap flow meter (Flow32-1 k, Dynamax, Houston, United States). Briefly, the area of the stem was calculated, and then the stem was wrapped in sap flow sensors to quantify its sap flow rate. The net photosynthesis and transpiration rates were measured between 9:00 am and 14:00 pm using a portable photosynthesis analyzer (LI-6400, LI-COR, Nebraska, United States). In the US treatment plots, some seedlings withered or died before March 2016, and almost all seedlings died by December 2016, owing to the lack of a host. Because of the limited number of plants remaining in the US treatment, no measurements were taken after September 2016.

### Sampling

Three plants were randomly selected from each plot for destructive sampling at 3-month intervals from March 2015 through March 2017. Lignified stems and roots (2–6 mm in diameter) were used for conducting a morphological analysis and measuring the starch content. Different types of haustoria (including pre-haustoria, post-haustoria, hasutoria attached to own roots, and post-haustoria attached to senescent host roots) were carefully identified and collected for morphological observation and starch quantification. Samples for morphological observation were cleaned, placed in formaldehyde, acetic acid, and ethanol (FAA) fixative, and stored at 4°C. Materials for determining starch levels were first thoroughly rinsed with running water and immediately dried in an oven for 2 h at 90°C. Then, these samples were dried at 65°C for 72 h and stored in a desiccator in the dark.

### Light Microscopy

Materials fixed in FAA were rinsed first with tap water and then with distilled water for 1 h. Then, stem, root, and haustorium sections were prepared using a new Gillette razor blade. Free-hand sections were stained with an iodine-potassium iodide (I-KI) solution or a solution of I-KI and phloroglucinol hydrochloride. These sections were observed and photographed under a stereomicroscope (M125; Leica, Germany).

Paraffin embedding and sectioning were conducted according to Zhou et al. (2008). Samples stored in the FAA fixative were rinsed with distilled water for 2 h. Then, stems and roots were cut into small (4–10 mm) pieces. These pieces and haustoria were dehydrated with an alcohol gradient (30, 60, 80, 95, and 100%) and then embedded in paraffin. The embedded materials were sectioned with a slicer (RM2235; Leica, Germany). The paraffin sections were baked in an oven at 59°C for 48 h, dewaxed, and rehydrated. The sections were then stained with Fast Green FCF and Safranin O. The stained sections were observed and photographed with a light microscope system (Eclipse E200; Nikon, Japan.).

### Scanning Electron Microscopy

The fixed samples were immersed in distilled water for 2 h to thoroughly wash off the fixative. The rinsed materials were cut into small pieces (2–5 mm long × 2–4 mm wide × 2–4 mm thick) appropriate for observation, while maintaining the characteristics of the material. These pieces were dehydrated using a gradient of alcohol and isoamyl isovalerate and then dried in a critical point dryer (DCP-1; Denton Vacuum, United States) using liquid CO_2_. The dried samples were glued to the upper surface of an aluminum stub with silver paste and then covered with gold powder in a gold sputter coating unit. Finally, the coated samples were observed under a scanning electron microscope (S-4800; Hitachi, Japan) and photographed.

### Measurement of Storage Starch and Soluble Sugar Content

Storage starch and soluble sugars were measured as described preciously (Chow and Landhäusser, 2004). All oven-dried samples were individually ground using a Mini Lab Planetary Ball Mill (MITR-YXQM-0.4 L; Miqi Instrument Equipment Co., Ltd., China) and passed through a 45-μm mesh sieve. The sieved powder (50 mg) was transferred into a centrifuge tube containing 5 ml of 80% ethanol. The tube containing the sample was placed in a 95°C water bath for 15 min and then centrifuged at 3,000 rpm for 5 min. The supernatant was transferred in a new test tube, and the residue was extracted twice using the method described above. The sample residue was stored at −20°C for subsequent determination of the starch content, while the supernatants of all three extractions were pooled and mixed thoroughly. Then, 500 μl of the mixed extract was transferred into a colorimetric tube. Later, 1 ml of 2% phenol and 2.5 ml of concentrated sulfuric acid were added to the extract, and the reaction was first incubated in the dark for 10 min and then placed in a 22°C water bath for 30 min. The absorbance of the sample was measured at 490 nm using a spectrophotometer (UV 2550, Shimadzu, Japan).

To measure the starch content, each sample residue was digested with an enzyme mix containing 1,500 U ml^−1^ *α*-amylase (cat. no. A4551; Sigma-Aldrich) and 20 U ml^−1^ amyloglucosidase (cat. no. ROAMYGLL; Sigma-Aldrich). This reaction system was incubated in a 50°C water bath for 24 h and then centrifuged at 3,000 rpm for 15 min. The supernatant was transferred into a new test tube and diluted with 0.05 M sodium acetate buffer; the volume of the buffer was dependent on the amount of the supernatant. Five hundred microliter of diluted sample solution was transferred into a colorimetric tube containing 2 ml of peroxidase-glucose oxidase (cat. no. P7119; Sigma-Aldrich) and *o*-dianisidine dihydrochloride (cat. no. C10362698; Shanhai Macklin Biochemical Co., Ltd.) reagent (PGO-color solution). The reaction system was incubated in the dark at room temperature for 45 min. Then, 500 μl of 75% sulfuric acid was added to the reaction system and mixed, and the absorbance of the solution was determined at 525 nm after 10 min. The starch concentration was determined by multiplying the glucose content with the conversion factor of 0.9 (Chow and Landhäusser, 2004). Sugar and starch contents of stems, roots, and haustoria were expressed as percentage dry matter (% DM).

### Statistical Analysis

All data, presented as mean ± SD, were first tested for normality and variance constancy. One-way ANOVA and the least significant difference (LSD) multiple comparison test were used to detect differences between the analyzed traits of different samples and at different determination times and between soluble sugar and starch content of various haustoria types. One-way ANOVA or paired *t*-test was used to test the differences between groups at the same sampling time. Differences were considered statistically significant at *p* < 0.05. SPSS 17.0 for Windows (SPSS, Inc., Chicago, IL, United States) was used to build the compound bar and multiline graphs and for conducting all statistical analyses.

## Results

### Effect of Different Treatments on the Growth of *S. album* Seedlings

**Figure 1:**
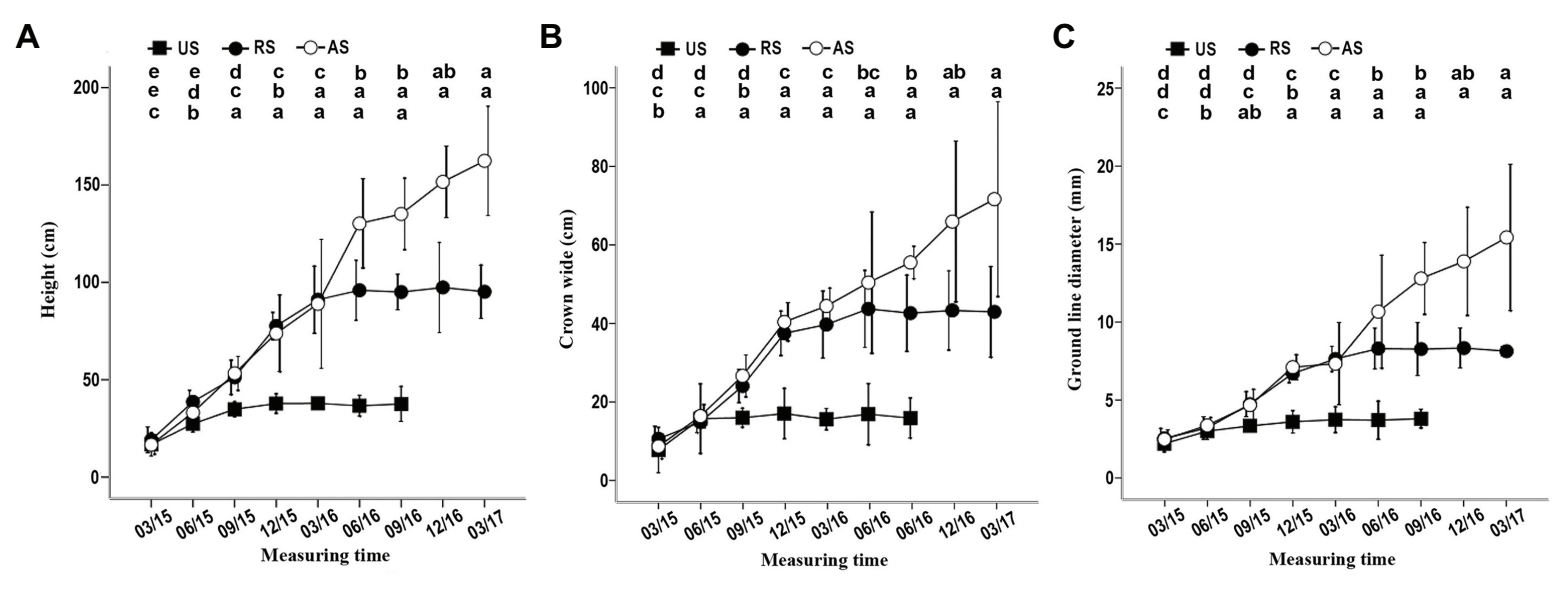
Effect of different treatments on the height, crown width, and ground line diameter of seedlings. **(A)** Effect of three treatments on the height. **(B)** Effect of three treatments on crown width. **(C)** Effect of three treatments on ground line diameter. In this and the following figures, error bars represent the standard deviation of three independent experiments, and 03/15, 06/15, 09/15, etc. indicate March 2015, June 2015, September 2015, etc. Different letters in the up, middle, and low rows of lowercase letters above each graph, respectively, indicate a statistical differences between measured values of seedlings of the AS, RS, and US treatments at different durations of the experiment. US, unattached seedling; AS, attached seedling; RS, host-removed seedling.

When *K. rosmarnifolia* seedlings (host) were transplanted near an *S. album* seedling (parasitic plant) in early February 2015, successful parasitization of the host seedling roots by *S. album* was observed after 10–20 days. In the first 4 months after planting, *S. album* seedlings in the US treatment showed similar growth indicators as those in the AS and RS treatments (Figure 1). However, after September 2015, the height, crown width, and ground line diameter of *S. album* seedlings in the US treatment decreased compared with the other treatments, and these gaps between US and AS or RS gradually increased with time (Figure 1). In September 2015, seedlings in the US treatment stopped growing and then slowly died between March and December 2016. By contrast, seedlings in the AS treatment showed a rapid increase in growth after September 2015 (Figure 1). The development of *S. album* seedlings in the RS treatment immediately slowed after the host plants were removed in November 2015 (Figure 1). After June 2016, the growth of *S. album* seedlings in the RS treatment stopped (Figure 1). Seedlings in the US treatment survived for approximately a year, although they had no hosts.

### Allocation Dynamics of Stored Starch and Soluble Sugar in the Stems and Roots of *S. album* Seedlings

**Figure 2:**
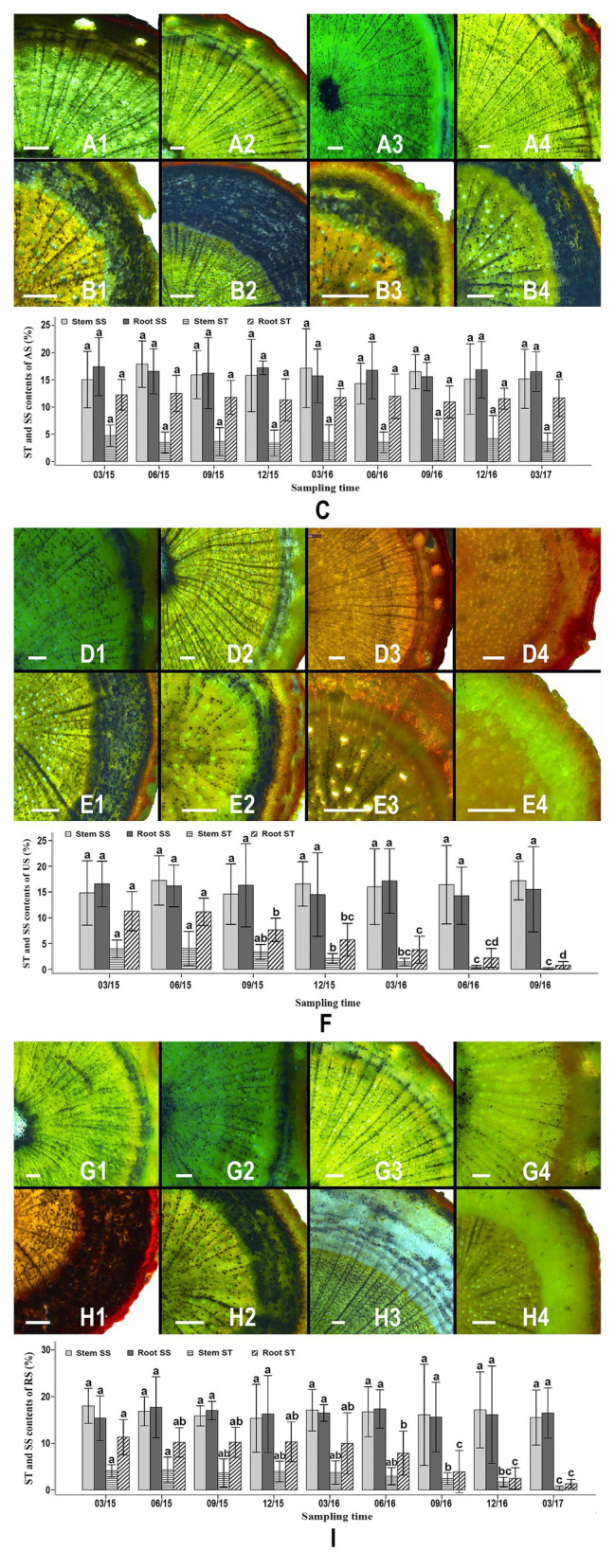
Starch granule distribution, starch levels, and sugar levels in the roots and stems of different seedlings over time. Allocation of starch granules in the stems of attached seedlings in March 2015 **(A1)**, September 2015 **(A2)**, June 2016 **(A3)**, and December 2016 **(A4)**. Allocation of starch granules in the roots of attached seedlings in March 2015 **(B1)**, September 2015 **(B2)**, June 2016 **(B3)**, and December 2016 **(B4)**. Variation of starch and soluble sugar contents in the stems and roots of attached seedlings between March 2015 and March 2017 **(C)**. Allocation of starch granules in the stems of unattached seedlings in March 2015 **(D1)**, September 2015 **(D2)**, March 2016 **(D3)**, and September 2016 **(D4)**. Allocation of starch granules in the roots of unattached seedlings in March 2015 **(E1)**, September 2015 **(E2)**, March 2016 **(E3)**, and September 2016 **(E4)**. Variation of starch and soluble sugar content in the stems and roots of unattached seedlings between March 2015 and September 2016 **(F)**. Allocation of starch granules in the stems of host-removed seedlings in March 2015 **(G1)**, September 2015 **(G2)**, June 2016 **(G3)**, and December 2016 **(G4)**. Allocation of starch granules in the roots of host-removed seedlings in March 2015 **(H1)**, September 2015 **(H2)**, June 2016 **(H3)**, and December 2016 **(H4)**. Variation of starch and soluble sugar content in the stems and roots of host-removed seedlings between March 2015 and March 2017 **(I)**. Starch granules in all sections were stained using iodine-potassium iodide (I-KI) to display black dots. AS, attached seedling; US, unattached seedlings; RS, removed seedling; ST, starch; SS, soluble sugar. In **(C,F)** and **(I)** different letters above error bars indicate a statistical difference (*p* < 0.05) between the starch and soluble sugar contents of stems or roots at different durations of the experiment. Scale bars = 200 μm in all sections.

Starch granules were abundant in the stem and root samples of *S. album* seedlings in the AS treatment throughout the study period (Figures 2A1–4,B1–4). In the AS treatment, the cortex and the phloem of seedling roots contained a greater amount of starch than the xylem (Figures 2B1–4). Starch granules were commonly observed in xylem rays, phloem rays, and cortex cells (Figures 2A1–4,B1–4). The starch contents of roots (10.943–12.515%) was higher than those of stems (3.385–4.745%; Figure 2C). No significant difference was observed in the starch content in the stems or roots of AS seedlings at various times, indicating that these starch levels did not correlate with the duration of the experiment (Figure 2C). The soluble sugar contents of stems and roots were higher than the starch contents of these tissues and accounted for 14.307–17.881% of the tissue dry weight. No significant difference was detected in the soluble sugar content of stems or roots of different AS seedlings, indicating that the soluble sugar levels were stable throughout the experiment (Figure 2C).

In the US treatment, abundant starch granules were observed in the stems and roots of *S. album* seedlings at the early stages (Figures 2D1,D2,E1,E2,F). The phloem and the cortex of roots contained more starch granules than the xylem (Figures 2D1,D2,E1,E2). However, the starch content of stems and roots of *S. album* seedlings in the US treatment declined after September 2015 (Figures 2D1–4,E1–4,F). The starch content of stems and roots of US seedlings decreased continuously from September 2015 to September 2016 until the content was less than 1%. The amount of starch in the stems of US seedlings before September 2015 was significantly greater than that after December 2015 (*p* < 0.05; Figure 2F). The levels of starch in the roots of US seedlings before June 2015 were significantly higher than those after September 2015 (*p* < 0.05; Figure 2F). By contrast, the soluble sugar content of stems or roots showed no significant difference between different US seedlings, which was similar to the results of the AS treatment (Figures 2C,F). In the RS treatment, seedlings stems and roots contained abundant starch prior to June 2016, similar to the seedlings in the AS treatment (Figures 2C, G1–4,H1–4,I). However, after the host plants were removed from the RS treatment in December 2015, the starch content of *S. album* seedlings started to decline in September 2016, reaching 0.398% in stems and 1.351% in roots by March 2017 (Figure 2I). The starch amount of roots of RS seedlings before June 2016 was significantly greater than that after September 2016 (Figure 2I). The starch contents of stems of RS seedlings after December 2016 were significantly lower than those before September 2016 (Figure 2I). Similar to the AS and US treatments, the RS treatment also showed no significant difference in the soluble sugar content of the stems or roots, thus indicating a stable trend throughout this experiment (Figures 2C,F,I).

### Allocation Dynamics of Starch and Soluble Sugar in Haustoria

**Figure 3:**
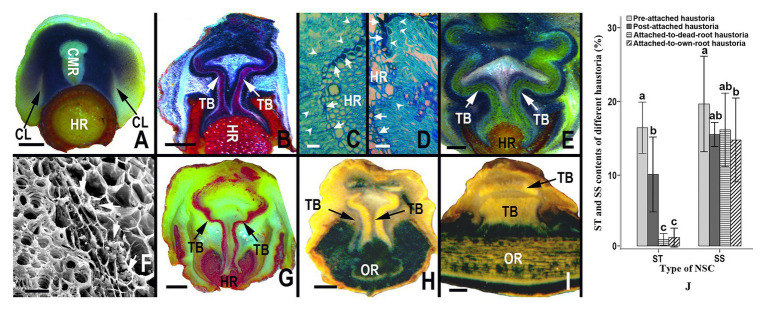
Starch granule distribution, and starch and sugar content in different haustoria. **(A)** Allocation of starch in pre-penetration haustoria, showing abundant starch granules between collapsed layers and the core meristematic region. **(B)** Allocation of starch granules in a haustorium penetrating a host root, showing rich starch granules in both sides of the tracheid bundles and sucker front zones. **(B,D)** Allocation of starch granules in the digital cells of haustorium at the parasite-host interface, showing that the digital cells containing more abundant starch granules (white arrowheads) can produce more secretory substance (white arrows). **(E)** Allocation of starch granules in a mature haustorium, showing rich starch granules in both sides of the tracheid bundles. **(F)** Abundant starch granules (white arrows) in several layers of parenchyma cells outside the tracheid bundle of a mature haustorium. **(G)** Allocation of starch granules in an inactive haustorium owing to the senescence or death of the host root, showing a lack of starch. **(H,D)** Allocation of starch granules in a haustorium attached to its own root, showing almost no starch particles on both sides of tracheid bundles in a transversal section and a longitudinal section. **(J)** Starch and soluble sugar content from different types of haustoria. In **(A,B)** and **(E–H)** the starch granules were stained by an I-KI solution to display black or blue-black. In **(C,D)** the starch granules were stained by Fast Green FCF to appear light blue-green, and the secretory substance were stained by Fast Green FCF to emerge dark-blue. The starch granules in **(A,B,E,G,I)** were stained by I-KI to appear dark-blue. In **(B,F)** the tracheid bundles were stained by the phloroglucinol solution to appear red. In **(A,E)** and **(G,H)** the tracheid bundles appear yellow. In **(A–H)** haustoria and roots were sectioned longitudinally and transversely, respectively; however, both the haustorium and the root were sectioned longitudinally in **(I)**. NSC, non-structural carbohydrate; ST, starch; SS, soluble sugar; CMR, core meristematic region; CL, collapsed layer; TB, tracheid bundle; HR, host root; OR, own root. In **(J)** different letters on error bars mean significant differences (*p* < 0.05) in analyzed starch or soluble sugar contents among different haustoria. Scale bars = 1 mm in **(A,B,E)** and **(G–I)**; 25 μm in **(C,D,F)**.

The allocation dynamics of starch in haustoria varied with the developmental stage of the haustoria and host roots; however, the soluble sugar content almost showed no significant difference among the different types of haustoria. At the early developmental stage, when haustoria had not yet begun to penetrate a root, starch granules were abundant in the zone between the collapsed layers and core meristematic regions (Figure 3A). While the haustorium was penetrating the cortex and the phloem of a root, starch granules were mainly concentrated on both sides of the tracheid bundles, as well as in the region between tracheid bundles in the interrupted zone (Figure 3B), and flange parenchyma cells on the sucker tip secreted some substances that stained dark-blue by Fast Green FCF (Figures 3C,D). The amount of substance secreted was directly proportional to the number of starch granules in these cells (Figures 3C,D). After successful attachment on the host root, the starch in the haustorium was still mainly distributed on both sides of the tracheid bundle and the area between vascular bundles in the interrupted zone (Figures 3E,F). However, the starch levels decreased when the haustorium did not absorb water and nutrients from a senescent or dead host root (Figure 3G). Haustoria attached to the roots of *S. album* contained fewer starch granules than those attached to the host roots (Figures 3H,I). The starch content in haustoria also showed that starch accounted for 16.437% DM of pre-attachment haustoria and 10.101% DM of post-attachment haustoria (Figure 3J). However, starch accounted for only 1.262% DM of post-attachment haustoria, when the host root was senescent or dead (Figure 3J). Starch content also accounted for only 1.551% DM of a post-attachment haustoriun, when attached to the root of *S. album* (Figure 3J). The results indicate that starch may be involved in the development and penetration of haustoria as well as in the absorption of water and nutrients from the host. Although the soluble sugar content of a pre-attached haustorium was significantly greater than that of a post-attached haustorium attached to the root of *S. album* (*p* < 0.05), there was no significant difference in the soluble sugar content among the other types of haustoria (*p* > 0.05; Figure 3J).

### Sap Flow, Net Photosynthesis, and Transpiration Rates of *S. album* Seedlings in Different Treatments

**Figure 4:**
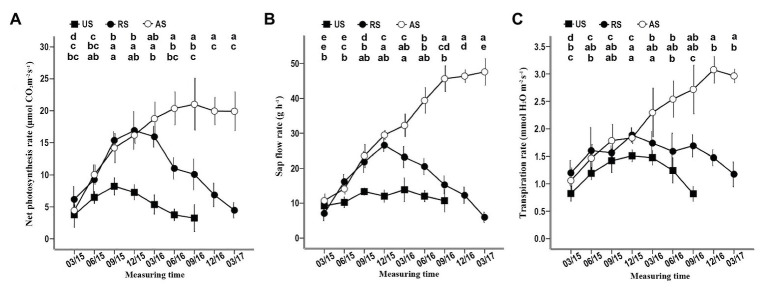
Effect of different treatments on the net photosynthesis, the sap flow rate, and the transpiration rate of seedlings. **(A)** Effect of different treatments on the net photosynthesis rate. **(B)** Effect of different treatments on the sap flow rate. **(C)** Effect of different treatments on the transpiration rate. Different letters in the up, middle, and low rows of lowercase letters above each graph, respectively, indicate a statistical difference between measured values of seedlings of the AS, RS, and US treatments in different duration of the experiment. US, unattached seedling; AS, attached seedling; RS, host-removed seedling.

In the AS treatment, the net photosynthesis, sap flow, and transpiration rates gradually started increasing in March 2015, reaching or approaching the maximum before December 2016 (Figure 4). When the hosts of RS seedlings were removed in December 2015, these three physiological indicators, especially sap flow and the net photosynthesis rate, gradually declined (Figure 4). In the US treatment, the sap flow, the net photosynthesis rate, and the transpiration rate increased in the first 9 months of 2015 (Figure 4), although these seedlings did not have hosts that provided water and nutrients. At the end of 2015, the net photosynthesis and transpiration rates began to decrease until the plants died, but the sap flow rate remained stable before June 2016 (Figure 4). These data confirm that successful attachment to the host root can significantly improve the physiological activity of *S. album* seedlings, promoting their growth and development.

### Morphological Characteristics of Starch Granules in Various Non-photosynthetic Vegetative Organs of *S. album*

**Figure 5:**
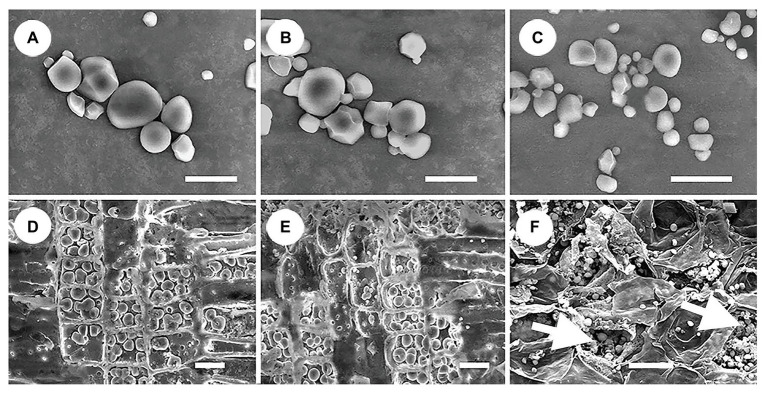
Shape and size of starch granules in different organs. **(A)** Starch granules in stems showing diverse shapes. **(B)** Starch granules in roots showing diverse shapes, and similar in size to those in the stems. **(C)** Starch granules in haustoria showing diverse shapes, and similar in shape to those of the stems, but smaller. **(D)** Dense arrangement of starch granules in the ray parenchyma cells in stems, showing little cytoplasm. **(E)** Dense arrangement of starch granules in the ray parenchyma cells in roots showing little cytoplasm. **(F)** Arrangement of starch granules in the parenchyma cells in haustoria showing abundant cytoplasm (white arrows). Scale bars = 10 μm in **(A–C)**; 20 μm in **(D–F)**.

All storage starch granules were distributed in the parenchyma cells of stems, roots, and haustoria. These granules exhibited a wide variety of shapes, such as spherical, hemispherical, tetragonal, polygonal, and irregular shaped (Figure 5). The diameter of starch granules in the three organs varied from 0.3 to 8 μm. Starch granules in stems and roots were similar in size, whereas those in haustoria were smaller (Figure 5). In addition, haustoria stored a greater number of small starch granules than stems and roots. The amount of cytoplasm was abundant in the haustorium parenchyma cells but scarce in the stem and root parenchyma cells (Figure 5). These differences may be related to the differences in the roles of these cells.

## Discussion

Previous studies confirmed that storage starch serves as an energy source for the sprouting, extension, and thickening of branches at the early growth stages of autotrophic plants (Bollmark et al., 1999; Kaelke and Dawson, 2005; Regier et al., 2010). In fire-prone plants of the Savanna and Mediterranean, roots of resprouters often contain much higher amounts of starch than those of seeders (Bell et al., 1996; Verdaguer and Ojeda, 2002; Knox and Clarke, 2005; Schutz et al., 2009; Wigley et al., 2009). In some perennial plants frequently consumed by herbivores, the stored starch provides energy for resprouting after damage to the aboveground organs (Hoffmann et al., 2004; Dietze et al., 2014). In the present study, the stems and roots of *S. album* seedlings in the AS, US, and RS treatments were rich in starch at the early stages of development. However, the starch content of US seedlings started declining from September 2015 onward until the plants died. The storage starch content of seedlings in the RS treatment also decreased when the host plants were removed. By contrast, the starch levels of AS seedlings remained stable throughout the experiment. As described above, plants disturbed by frequent external stresses usually stored more starch than their undisturbed counterparts did. Starch provides energy and substances to plants under stress conditions, such as low temperature, drought, wildfire, and disturbance. In this context, the lack of a host may be considered an external stressor to the hemiparasitic tree; therefore, it is reasonable that a vital role of storage starch is to supply energy and substrates for *S. album* seedlings under conditions where no host is present.

The study of Barrett and Fox (1997) showed that, 3 months after seedling emergence, the height and the leaf number in unattached *S. album* seedlings stopped increasing in a no-nutrient substrate. However, seedlings grown in an all-nutrient substrate continued increasing in height and leaf number, suggesting that these seedlings obtain water and nutrients from the substrate *via* their roots at the early stage of development (Barrett and Fox, 1997). In the current study, the height and crown width of *S. album* seedlings in the US treatment gradually stopped increasing after 6 months. Seedlings in the US treatment accumulated a similar amount of starch as seedlings in the AS treatment during the first several months of growth. These results support the speculation of Barrett and Fox (1997) that seedlings in the US treatments can acquire nutrients from the seed storage reserve and *via* their roots from the cultivation substrate at an early developmental stage.

Previous studies on *S. album* and *C. japonica* seedlings showed that abundant starch granules are accumulated in their haustoria at an early developmental stage (Lee, 2007; Zhang et al., 2012; Yang et al., 2014). Yang et al. (2014) found that the collapsed layers are developed from starch-containing cells that surround the vascular meristematic region. Numerous secretory granules are also detected in these starch-containing cells. Zhang et al. (2012) reported abundant starch granules are present during the development of haustoria, particularly in the outermost cells located next to the endophytic tissue of a haustorium. These authors also showed that the finger-shaped parenchyma cells situated near the haustorium-host interface produced a secretory substance, which potentially degrades the walls of host roots (Zhang et al., 2012). In addition, the amount of secretory substances secreted by the finger-shaped parenchyma cells seemed to depend on the starch content, indicating that starch may be involved in the development of haustoria, as postulated by some researchers (Yang et al., 2014). The present study supported the previous speculation that starch in the endophytic tissue, especially in finger-shaped cells, may be associated with the production of secretory substances disintegrating the walls of host roots and the insertion of a haustorium penetrating into a root. Our results also showed that abundant starch granules at both the inner and outer sides of vascular cells of the vascular core in a healthy, post-attached haustorium. However, few starch granules were detected in the same regions of an inactive haustorium, which performed no function because of the senescence or death of a host roots. Therefore, it demonstrates that starch may also be involved in the transport of water and nutrients from host roots to the haustoria.

Some studies reported that parasitic plants have a lower water potential than their hosts (Smith and Stewart, 1990; Ackroyd and Graves, 1997; Jiang et al., 2005; Cameron and Seel, 2007; Yoshida et al., 2016). Osmotically active compounds, such as sugars and sugar alcohols, decrease the water potential of cells (Hodgeson, 1973; Tennakoon et al., 1997; Jiang et al., 2005; Brokamp et al., 2012; Těšitel et al., 2015). In plants, soluble sugar or osmosis-related substances can be supplemented by the degradation of storage starch under stress conditions or in the absence of photosynthesis (Sauter, 1988; Barbaroux and Bréda, 2002; Chaves et al., 2003; Lee et al., 2008; Salleo et al., 2009; Galvez et al., 2011; Hasibeder et al., 2015; Li et al., 2018; Wang et al., 2018). In the current study, although the starch content of the stems and roots of *S. album* seedlings in the US or RS treatment decreased, the soluble sugar content remained stable. Therefore, storage starch may supplement osmotic substances in the US and RS treatments through a degradation process, thus contributing to maintenance of a low water potential in *S. album*.

In autotrophic plants, storage starch plays a crucial role in providing energy for growth and development and in offering active compounds for osmotic regulation under stress conditions. Consistent with previous studies (Zhang et al., 2012; Yang et al., 2014), our results suggest that starch may be involved in the initiation and development of haustoria of *S. album*. The results also indicate that storage starch of *S. album* may not only provide energy and osmosis-related substances when the hemiparasitic tree lose its host but also be related to the physiological activities of haustoria, such as root penetration, water absorption, and nutrient absorption. From an evolutionary perspective, storage starch in *S. album* may have acquired novel roles under selective pressure. Overall, this study provides a comprehensive understanding of the roles of starch in parasitic plants.

## Data Availability Statement

All datasets generated for this study are included in the article/supplementary material.

## Author Contributions

XZ, NZ, and DX conceived the project and designed the experiments. NZ, LD, GX, and JT performed the experiments and analyzed the data. XZ and YZ drafted the manuscript. All authors contributed to the article and approved the submitted version.

### Conflict of Interest

The authors declare that the research was conducted in the absence of any commercial or financial relationships that could be construed as a potential conflict of interest.
